# Recurrence of anti-MDA5 antibody-positive clinically amyopathic dermatomyositis after long-term remission: A case report

**DOI:** 10.1097/MD.0000000000011024

**Published:** 2018-06-29

**Authors:** Yushiro Endo, Tomohiro Koga, Midori Ishida, Yuya Fujita, Sosuke Tsuji, Ayuko Takatani, Toshimasa Shimizu, Remi Sumiyoshi, Takashi Igawa, Masataka Umeda, Shoichi Fukui, Ayako Nishino, Shin-ya Kawashiri, Naoki Iwamoto, Kunihiro Ichinose, Mami Tamai, Hideki Nakamura, Tomoki Origuchi, Masataka Kuwana, Yuji Hosono, Tsuneyo Mimori, Atsushi Kawakami

**Affiliations:** a Department of Rheumatology, Unit of Advanced Preventive Medical Sciences, Graduate School of Biomedical Sciences, Nagasaki University, Nagasaki; b Department of Allergy and Rheumatology, Nippon Medical School Graduate School of Medicine, Tokyo; c Department of Rheumatology and Clinical Immunology, Graduate School of Medicine, Kyoto University, Kyoto, Japan.

**Keywords:** anti-MDA5 antibody, clinically amyopathic dermatomyositis, dermatomyositis, interstitial pneumonia

## Abstract

**Rationale:**

Among all dermatomyositis (DM) patients, antimelanoma differentiation-associated gene 5 antibody (anti-MDA5 Ab) positive patients have significantly poor short-term mortality, whereas they experience less relapses over the long term after the remission. We report the case of a patient with anti-MDA5 Ab-positive clinically amyopathic dermatomyositis (CADM) with the recurrence of interstitial lung disease (ILD) after 7 years of remission. There has been no case report of an anti-MDA5 Ab-positive DM patient with the recurrence of ILD after 7 years of long-term remission.

**Patient concerns:**

A 70-year-old Japanese woman was diagnosed with anti-MDA5 Ab-positive CADM and ILD. After achieving 7 years long-term remission, she was admitted to our department with erythema on the fingers and interstitial pneumonia. Her anti-MDA5 Ab titer was elevated.

**Diagnoses:**

We diagnosed recurrent CADM complicated with ILD.

**Interventions:**

We successfully treated her with 1,000 mg of methyl-prednisolone pulse and intravenous cyclophosphamide therapy followed by prednisolone 50 mg/day and an increase of cyclosporine.

**Outcomes:**

After that treatment, the patient's skin symptoms and interstitial pneumonia were relieved. All laboratory investigations such as ferritin, the serum markers of interstitial pneumonia (i.e., SP-A, SP-D), and the titer of anti-MDA5 Ab showed signs of improvement.

**Lessons:**

Her case suggests that careful physical examinations and monitoring the serum markers are important even after long-term remission is achieved.

## 1 Introduction

Dermatomyositis (DM) characterized by amyopathy or hypomyopathy with the typical skin symptoms is defined as clinically amyopathic dermatomyositis (CADM).^[1]^ Among DM patients, antimelanoma differentiation-associated gene 5 antibody (anti-MDA5 Ab) has been identified as a new autoantibody.^[2,3]^ It has been widely recognized that anti-MDA5 Ab-positive DM patients frequently develop rapidly progressive interstitial lung disease (RPILD) with poor prognosis.^[2,4]^

Among all DM patients, the anti-MDA5 Ab-positive patients have significantly poor short-term mortality over the first 6 months after diagnosis, whereas no significant difference in the long-term mortality over the first 2 years post-diagnosis was observed between anti-MDA5 Ab-positive patients and anti-MDA5 Ab-negative patients, suggesting that anti-MDA5 Ab-positive patients experience less relapses over the long term.^[5]^ However, there have been no sufficient studies of the long-term prognosis and relapse rate of anti-MDA5 Ab-positive DM patients after remission.

We herein report the case of a patient with anti-MDA5 Ab-positive CADM with the recurrence of interstitial lung disease (ILD) after 7 years of remission, treated successfully by a combination of corticosteroids, cyclophosphamide and calcineurin inhibitor.

## 2 Case report

In October 2010, a 70-year-old Japanese woman was diagnosed with ILD and CADM based on the findings of rash on the fingers of both hands, interstitial pneumonia, and a high titer of anti-MDA5 Ab (148 index). We administered 1000 mg of methyl-prednisolone (mPSL) pulse therapy and intravenous cyclophosphamide therapy (IVCY) followed by prednisolone (PSL) 50 mg/day with tapering and cyclosporine (CyA), and the symptoms were improved by a total of 5 IVCY continuations. After that, the patient maintained long-term remission for approximately 7 years, and she was treated with oral PSL 3 mg/day and CyA 100 mg/day. However, in November 2017, she suffered from nasal discharge, feeling heaviness of her head, and fatigue. In December 2017, she also presented with rash on both her fingers and toes, and she was then admitted to our department.

**Figure 1:**
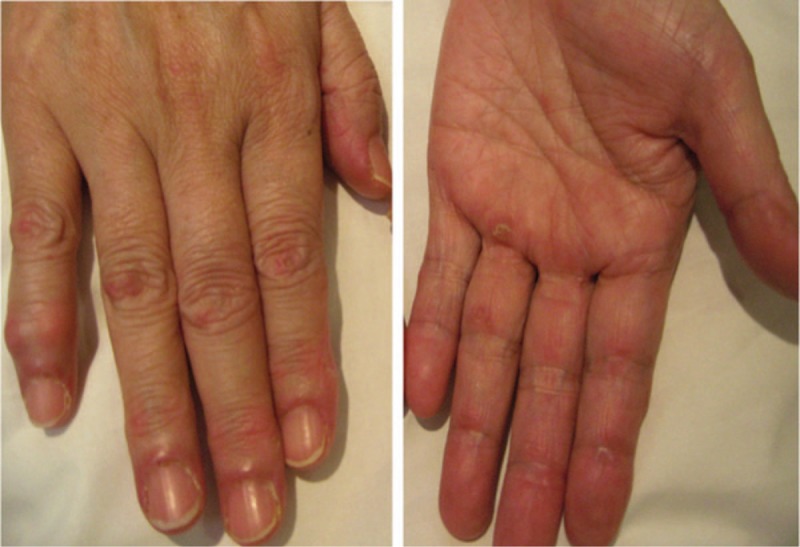
Erythemas on the nail circumference and both dorsal and palm sides around the PIP and MCP joints. MCP = metacarpophalangeal (MCP), PIP = proximal interphalangeal.

On admission, her body temperature was 37.1°C; her blood pressure was 141/76 mm Hg, the heart rate was 86 beats/min, and the pulse oximetric saturation (SpO_2_) was 95% (room air). On physical examination, fine crackles were audible on the dorsal side of the bilateral lower lung regions, and she had erythemas on the nail circumference and both dorsal and palm sides around the proximal interphalangeal (PIP) and metacarpophalangeal (MCP) joints, suggesting Gottron's sign and inverse Gottron's sign, respectively (Fig. 1). She had no muscle pain, and a manual muscle test showed no abnormalities in the upper and lower limbs.

Laboratory investigations showed the following results: partial pressure of arterial oxygen (PaO2) 73.5 mm Hg, white blood cell count (WBC) 9400 /μL (neutrophils 89.5%, lymphocytes 9.3%), hemoglobin (Hb) 12.1 g/dL, platelet (PLT) 27.0 × 10^4^/μL, C-reactive protein (CRP) 3.4 mg/dL, lactate dehydrogenase (LDH) 231 IU/mL (normal range 124–222), ferritin 319 ng/mL (normal range 6.0–138). The levels of creatinine kinase and aldolase were 79 IU/L and 3.6 IU/L, respectively (= within the normal range). Although the serum Krebs von den lungen (KL)-6 level was 274 U/mL (within the normal range), the levels of surfactant protein (SP)-A and SP-D were 49.3 and 140 ng/mL in slightly high titers. No abnormalities were revealed by a urinalysis, and no liver or renal dysfunction was detected.

**Figure 2:**
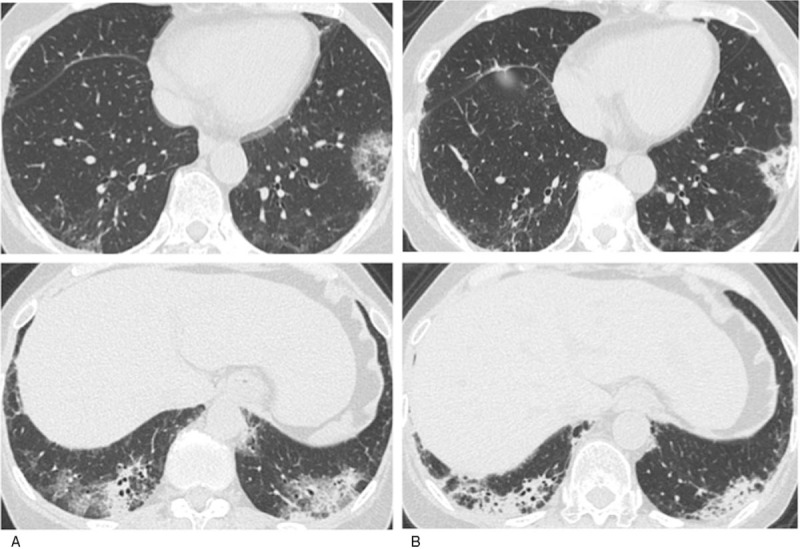
A chest CT on admission showed the expression of invasive shadows on lung field under the pleura and on the dorsal side of the bilateral lower lobes (A), after strengthening treatment it showed contraction of invasive shadows (B).

The following immunological and serological results were all negative (the exception is anti-MDA5 antibody): rheumatoid factor (RF), antinuclear antibody (ANA), proteinase-3 anti-neutrophil cytoplasmic autoantibodies (PR3-ANCAs), myeloperoxidase anti-neutrophil cytoplasmic autoantibodies (MPO-ANCAs), anti-ARS antibody, anti-transcription intermediary factor 1-gamma (TIF1-γ) antibody, and angiotensin converting enzyme (ACE). The anti-MDA5 antibody titer index was 109 (normal range < 32). The results of assays of β-D-glucan, T-SPOT. TB *Legionella pneumophila*, *Streptococcus pneumonia*, *Mycoplasma pneumonia*, *Clamydia psittaci*, and *Clamydia pneumonia* were all negative. A chest computed tomography (CT) examination showed the expression of invasive shadows on lung field under the pleura and on the dorsal side of the bilateral lower lobes (Fig. 2A), suggesting an exacerbation of interstitial pneumonia.

**Figure 3:**
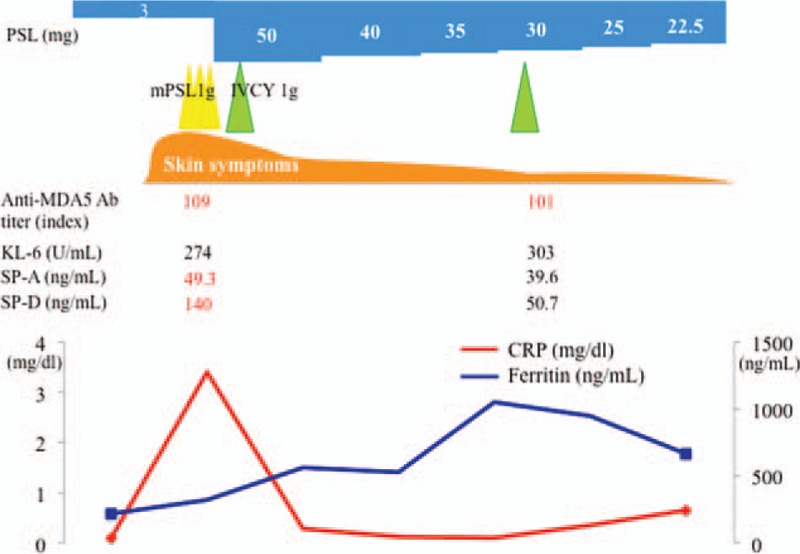
The clinical course of the patient, a 70-year-old Japanese woman. The CRP, ferritin level, and the treatment interventions during the hospital course are shown. CRP = C-reactive protein, IVCY = intravenous cyclophosphamide therapy, mPSL = methyl-prednisolone.

We diagnosed the patient as having recurrent CADM complicated with ILD based on the findings of the typical skin symptoms, the exacerbation of interstitial pneumonia, and a high titer of anti-MDA5 Ab. We then administered 1,000 mg of mPSL pulse therapy and IVCY followed by PSL 50 mg/day with tapering and an increase of CyA from 100 mg to 150 mg. After that treatment, the patient's skin symptoms and interstitial pneumonia were relieved (Fig. 2B). All laboratory investigations such as ferritin, the serum markers of interstitial pneumonia (i.e., SP-A, SP-D), and the titer of anti-MDA5 Ab showed signs of improvement. We administered a total of 2 sessions of IVCY continuation, and the patient's remission has now been maintained for over 1 months as of this writing (Fig. 3). We measured the antisplicing factor proline/glutamine-rich protein antibody (anti-SFPQ Ab) using her preserved serum and found that anti-SFPQ Ab at the initial diagnosis was negative, but it turned positive at the recurrence.

## 3 Discussion

We treated an anti-MDA-5 Ab-positive patient with recurrent CADM complicated by ILD who had maintained long-term remission for approximately 7 years after the initial diagnosis of the disease. Her case provides significant information about the mechanisms underlying the onset of ILD, the long-term prognosis, and the treatment strategies after the remission among anti-MDA-5 Ab-positive patients.

Melanoma differentiation-associated gene 5 (MDA5), which is the target autoantigen against anti-MDA5 Ab, belongs to the retinoic acid-inducible gene I (RIG-I) family and plays important roles in the innate immune system during virus infections through antiviral cytokines such as type I interferon (IFN) and tumor necrosis factor-alpha (TNF-α).^[6,7]^ Each protein molecule in the RIG-I family recognizes a different type of virus, and MDA5 is a molecule necessary for recognizing picornaviruses including coxsackievirus,^[8]^ suggesting that the production of anti-MDA5 Ab and the onset of CADM complicated with ILD are autoimmune phenomena induced by viral infection.^[3,9]^

In order to fully understand the clinical symptoms and prognosis of anti-MDA5 Ab-positive cases, it is necessary to consider differences among races and geographic regions. Japanese reports of anti-MDA5 Ab-positive DM patients demonstrated the following prevalences: CADM, approximately 80%; ILD, approximately 90%; RPILD, approximately 70%; and mortality, approximately 30%–50%,^[3,9–13]^ indicating anti-MDA5 Ab-positive DM patients have poor prognoses. In addition, according to the reports from other East Asian countries, there was no significant difference in the prevalence of RPILD or the mortality rate between these countries and Japan. However, most of those reports showed that the prevalence of CADM is ≤40% in other East Asian countries, suggesting that this prevalence of anti-MDA5 Ab-positive CADM is much lower than that of Japan.^[14–17]^ In North America, the prevalence of CADM is approximately 50%, whereas the prevalence of RPILD is approximately 20%, indicating that the prevalence of RPILD with anti-MDA5 Ab-positive is much lower than that of Japan.^[18,19]^

These racial and regional differences may be due to genetic backgrounds and environmental factors. The frequency of anti-MDA5 antibody-positive cases was reported to be higher around the Kiso River in Japan,^[20]^ suggesting environmental involvement. There are also several reports that anti-MDA5 antibody-positivity is more frequent among individuals with HLA-DRB1 gene polymorphism,^[21–23]^ which suggests genetic involvement. Although the mechanisms underlying the onset of ILD in anti-MDA5 Ab-positive DM patients have not been elucidated, the case of our present patient (who experienced 2 episodes of onset) suggests a genetic factor rather than an environmental factor.

Our patient's case showed that serum anti-SFPQ Ab turned to be positive at the recurrence. Anti-SFPQ Ab is a new DM-specific autoantibody, which is particularly specific for anti-MDA5 Ab-positive DM.^[24]^ SFPQ is a multifunctional nuclear protein associated with the human gene expression pathway, RNA production and processing, and viral infection.^[24,25]^ Because both SFPQ and MDA5 have important roles in viral infection, the recurrent disease in our patient's case may suggest an association with viral infection. A previous report showed that there was no significant difference in the frequency of the recurrent between anti-SFPQ Ab-positive (n = 27) and -negative (n = 24) patients with anti-MDA5 Ab-positive DM, but all 5 anti-MDA5 Ab-positive patients who showed the recurrence were anti-SFPQ Ab-positive.^[24]^ Our patient's case may suggest an association between the recurrent and the appearance of anti-SFPQ Ab.

Measuring the anti-MDA5 Ab level is a novel tool for monitoring disease activity in RPILD with DM,^[26]^ and hyperferritinemia predicts poor prognosis, especially if the ferritin level is >1600 ng/mL.^[27]^ The anti-MDA5 Ab, ferritin, and IL-18 levels in the serum are also useful for the evaluation of response to treatment in ILD patients with anti-MDA5 Ab-positive DM.^[12]^ In other words, the anti-MDA5 Ab level is a novel parameter for monitoring and a good predictor of RPILD relapse in patients with DM.^[28]^ It was reported that 4 of 12 patients with RPILD relapsed with anti-MDA5 antibody-positive DM in both an anti-MDA5 antibody sustained-positive group and the negative conversion group; the same study showed that RPILD relapses are associated with a re-increase in anti-MDA5 Ab level in all patients (4/4), and also showed that patients with a sustained high titer of anti-MDA5 Ab tend to relapse earlier than those with who are negative for anti-MDA5 Ab.^[28]^ In our patient's case, the ferritin level during the remission after the initial treatment was sustained at a mildly high level at 200 ng/mL, but we did not monitor her anti-MDA5 antibody titer during the remission; we feel that from now on it is necessary to monitor the anti-MDA5 antibody titer as well as the ferritin level.

Although the recurrence of ILD among anti-MDA5 Ab positive-patients had been believed to be less until now, it is important in actual clinical practice to predict the recurrence at an early stage from clinical signs. Our patient presented no respiratory symptoms at the recurrence, but we suspected the onset of a recurrence because of the characteristics of her skin symptoms, leading to the early detection of ILD exacerbation. Accordingly, the careful examination on her skin symptoms was extremely important not only at the initial diagnosis but also at the evaluation of the recurrence.

As the initial treatment of RPILD with anti-MDA5 Ab-positive DM patients, the combination of corticosteroids, cyclophosphamide, and calcineurin inhibitor is recommended.^[29]^ However, there are no established treatment guidelines for the prevention of recurrence during remission. Our patient had been treated with oral administration of PSL 3 mg/day and CyA 100 mg/day during the remission, but her ILD eventually was recurred, accompanied by skin lesions. Anti-MDA5 Ab-positive DM patients had a lower relapse rate during the 2 years after the initial treatment compared to other DM patients.^[4,5]^ However, it has been only about 10 years since DM patients with anti-MDA5 antibody-positive were well recognized. Although our patient's case indicates the possibility that ILD can be recurred after long-term remission, further investigation is needed to clarify the long-term prognosis among anti-MDA5 Ab-positive DM patients.

In conclusion, we successfully treated an anti-MDA5 Ab-positive CADM patient with a recurrence of ILD after she achieved long-term remission (approximately 7 years). It is commonly recognized that anti-MDA5 Ab-positive DM patients have significantly poor short-term mortality, whereas the long-term prognosis of these patients is relatively good. However, we would like to emphasize the necessity of careful monitoring for the recurrence of ILD, even after a patient achieves long-term remission. Our patient's case suggests that careful physical examinations of skin symptoms as well as the serum markers including the titer of MDA5 Ab during the remission of ILD provide a chance to make an early diagnosis of the recurrence and early intensive treatment.

## Author contributions

**Investigation:** Yuji Hosono, Tsuneyo Mimori.

**Supervision:** Tomohiro Koga, Midori Ishida, Yuya Fujita, Sosuke Tsuji, Ayuko Takatani, Toshimasa Shimizu, Remi Sumiyoshi, Takashi Igawa, Masataka Umeda, Shoichi Fukui, Ayako Nishino, Shin-ya Kawashiri, Naoki Iwamoto, Kunihiro Ichinose, Mami Tamai, Hideki Nakamura, Tomoki Origuchi, Masataka Kuwana, Atsushi Kawakami.

**Writing – original draft:** Yushiro Endo.
